# Supplementary material for: Are carbon emissions trading and green financial instruments synergistic? -Comprehensive quantitative research based on content analysis
Source: PLoS One. 2024 Mar 7;19(3):e0298601. doi: 10.1371/journal.pone.0298601 (PMC10919601; doi:10.1371/journal.pone.0298601)
Supplement: S1 Data — (ZIP) [file pone.0298601.s001.zip › Data/地级市Z_F工作报告.zip!/╣π╢1⁄2/╔ε█┌╩╨/└┤╫╘╣1⁄2╓┌║┼í╛┬φ┐╦╩2╛▌═°í┐_2006.docx]

各位代表：

　　 现在，我代表深圳市人民政府向大会作政府工作报告，请予审议，并请各位政协委员和其他列席人员提出意见。

　　 一、“十五”工作简要回顾和2005年经济社会发展基本情况

　　 “十五”时期是深圳经济社会发展转型的重要时期。全市人民在党中央、国务院、省委、省政府和市委的正确领导下，以邓小平理论和“三个代表”重要思想为指导，认真落实科学发展观，明确了建设和谐深圳效益深圳和国际化城市的发展目标，创新思路，坚定信心，团结开拓，真抓实干，“十五”计划的主要指标超额完成，三个文明建设取得重大成就，经济社会开始全面转入新的发展轨道，成为首批“全国文明城市”。

　　 ——经济实力显著增强。按深圳市第一次全国经济普查口径计算，“十五”期间，深圳本市生产总值连续跨越3000、4000亿元台阶，达到4926.9亿元，年均增长16.3%。其中工业和第三产业增加值分别占49.4%和47.4%。按调整后的常住人口数计算，人均GDP达到7483美元。2005年全市外贸出口1015亿美元，成为全国首个出口超千亿美元的城市，外贸出口总额连续13年保持全国第一。社会消费品零售总额1437.7亿元，位居全国大中城市第四位。全口径财政收入1360亿元，其中地方财政一般预算收入达到412.4亿元。五年累计向中央财政贡献3500亿元，相当于“九五”时期的2.5倍。在国家统计局最新公布的全国综合实力百强城市排名中，深圳位居全国第三。

　　 ——城市功能更加完善。经济中心城市的辐射和服务功能增强，高科技城市、区域性金融中心城市、物流枢纽城市、滨海旅游城市以及文化—生态城市建设稳步推进。2005年高新技术产品增加值1400亿元，占本市生产总值28.4%，拥有自主知识产权的产品产值占高新技术产品总产值的58%。专利申请量突破2万件，在全国大中城市中位居第三，PCT国际专利申请超过700件，居全国大中城市第一位。“高交会”国际影响力不断扩大。物流业实现跨越式发展，深圳港集装箱吞吐量突破1600万标箱，在世界集装箱枢纽港的排名由2000年的第十一位跃升至第四位。深圳机场继续保持全国第四大航空港地位，旅客吞吐量突破1500万人次，货邮行吞吐量达到55万吨。金融业总资产突破1万亿元，深交所成功开设中小企业板，银行、保险、基金、创投业迅速发展，金融业综合实力和竞争力位居全国前列。成功举办首届深圳国际文化产业博览会，华侨城、大芬村成为全国文化产业示范基地，文化产业正在发展成为新的支柱产业。旅游环境不断完善，2005年接待游客总人数5782万人次，其中接待过夜国外游客120万人次，位居全国大中城市第四位。

　　 ——改革开放深入推进。行政管理体制改革不断深入，有限政府、责任政府、高效政府、法治政府和廉洁政府建设取得成效，机关作风进一步好转。国有资产管理体制进一步完善，国有企业改革和国有经济布局调整的阶段性任务基本完成，一批主导产业突出、核心竞争力强的大型产业集团健康发展，国有资产实现了安全保值和增值。民营经济保持良好发展势头，成为自主创新的主力军。投融资体制改革和公用事业建管体制改革取得成效，市场信用体系逐步建立，知识产权保护得到加强，社会管理、人口管理、事业单位管理、财政管理体制改革和文化体制改革等取得阶段性成果。积极应对加入WTO的新形势，开放环境不断优化。“十五”期间吸引外商直接投资累计147.24亿美元，世界500强企业已有114家投资我市。与香港签订“1＋8”合作协议，制定融入泛珠三角区域合作实施方案，与国内其他地区的合作更加密切。

　　 ——城市建设步伐加快。“十五”时期，全社会固定资产投资累计超过4500亿元。先后实施400多个重大项目，地铁一期、市民中心、会展中心等相继投入使用。西部通道和盐田、蛇口、大铲湾等港区新建扩建工程加快推进，“一横八纵”城市干线路网建设顺利实施，城市基础设施不断完善。东部供水水源工程、供水网络干线工程竣工。加强城市生态保护和建设，建成区绿化覆盖率达到45%。加速推进宝安、龙岗两区城市化进程，深圳成为全国首个没有农村的大城市。以对历史负责、对城市负责、对人民负责的态度，强力推进“净畅宁工程”、“梳理行动”、“查违”、“清无”和城中村改造等重大行动，城市面貌显著改观。先后荣获首届“中国人居环境奖”、联合国环境保护“全球500佳”、“国家卫生城市”、“国家绿化模范城市”、“国家园林城市”、“中国保护臭氧层贡献奖特别金奖”等称号。

　　 ——人民生活水平继续提高。2005年，居民人均可支配收入2.15万元。户籍居民登记失业率控制在2.37%的较低水平。社会保障体系日趋完善，覆盖面不断扩大，城市化人员全部纳入社保体系。医疗卫生服务体系不断完善，公共卫生设施和社区健康服务中心建设加快，有效抗击、防范了“非典”和高致病性禽流感等重大疫情。持续开展“关爱行动”，完善收入分配体系，切实解决国企改革分流人员和特殊困难群体的生产生活问题。对口帮扶和“同富裕工程”取得成效。

　　 ——社会文明全面进步。群众性精神文明创建活动成效显著，市民综合素质明显提高，郭春园、丛飞等先进人物成为新时代深圳精神的代表。文化事业加快发展，文艺精品不断涌现。教育投入持续增加，新增中小学学位38.6万个，成为全省首个教育强市。深圳大学成功申请成为新增博士学位授权单位，深圳职业技术学院等院校建设取得显著成绩，大学城西校区建成投入使用，高等教育初步实现跨越式发展。高度重视人才工作，人才结构进一步优化。深入实施固本强基工程，基层组织建设和社区建设进一步加强。依法治市深入推进，法治化进程明显加快。持久开展社会治安综合治理，获得“全国社会治安综合治理优秀地市”称号。广泛开展全民健身活动，在奥运会、全运会和省运会等重大体育赛事上获得优异成绩，第二十六届世界大学生运动会申办工作顺利进行。加强人口和计生工作，较好地完成了人口计生目标管理任务。加强民兵预备役建设和民防工作，连续五年荣获“全国双拥模范城”称号。统计、外事、侨务、港澳台务、民族宗教、档案、气象等各项事业都取得新进步。

　　 各位代表，刚刚过去的2005年，是本届政府的开局之年。我们认真贯彻市第四次党代会精神，按照市四届人大一次会议的决议要求，狠抓落实，本市生产总值增长15%，每平方公里土地生产总值和财政收入分别达到2.5亿元和7000万元，万元GDP建设用地、能耗和水耗继续下降，空气质量和河流水质有所改善，市四届人大一次会议确定的十项重点工作全面完成。

　　 （一）按照效益深圳的发展要求，全面推进产业结构优化升级。确立建设国家创新型城市的目标，推出了以自主创新推动经济社会发展的各项政策措施，制定和实施《深圳市落实科学发展观经济社会调控指标体系》。一批重大产业项目正在积极规划和实施。服装、家具、模具、黄金珠宝等优势传统产业集聚基地建设加快。盐田港保税物流园区通过封关验收，六大物流园区建设稳步推进。出台加快深圳金融业改革创新发展的若干意见，股权分置改革取得积极进展，中小企业板块成为全国第一个全流通板块，华南技术产权交易中心正式挂牌，上海黄金交易所深圳备份交易中心开业，深圳农联社成功改制，与香港、伦敦金融城的金融合作加强。出台大力促进文化产业发展的决定及相关配套政策，推动了文化产业的快速发展。商贸、旅游、会展等服务业保持良好发展势头，成功举办首届深圳国际旅游文化节。

　　 （二）高度关注民生，着力解决了一批市民关注的热点难点问题。大力加强食品安全工作，启动“五大工程”、“四大体系”建设，积极探索建立完整有效的食品安全监管体系，食品安全专项整治取得良好效果。开展工业区食堂整顿与建设，解决劳务工就餐难问题。大力实施治污保洁工程，福田河、新洲河、大沙河第一阶段整治工程完工，盐田河完成截污工程，四条河流中上游河段基本实现“不黑不臭”。继续加大机动车尾气、固体废弃物等污染治理。顺利完成国家生态环境监察试点工作，龙岗区成为全国生态示范区。帮助2.9万失业人员实现就业和再就业，2900多户“零就业家庭”实现每户1人以上就业。加强劳动保障监察和劳动争议协调处理，在全国首创行政和司法联动，重拳打击恶意欠薪，切实维护劳动者合法权益。政府投资项目工程款清欠和农民工工资清欠均100%完成。基本养老、基本医疗、失业保险、工伤、生育医疗保险五个险种参保人数均创历史新高。提高城市居民最低生活保障水平和劳动者最低工资标准，逐步扩大劳务工医疗保险范围，受惠劳务工达124万。医疗重点项目和社区医疗网点建设加快，市中医院住院楼、妇幼保健院大楼等即将交付使用，人民医院改扩建工程顺利推进，新增36家社区健康服务中心和120余家社区医疗服务站。

　　 （三）着力增强城市功能，重大基础设施建设加速推进。广深港客运专线广深段开工建设，厦深铁路建设前期工作进展顺利。西部港区新建4个泊位，新增集装箱吞吐能力150万标箱，大铲湾港区一期工程开工建设，盐田港区三期扩建工程顺利推进。深圳机场扩建陆域形成工程正式启动。龙大高速、清平快速路一期、南坪快速路一期西段建成通车，盐排高速、南光高速、深盐二通道、福龙路等工程进展顺利。轨道交通1号线和4号线续建工程试验段、3号线土建暨深惠路改扩建工程开工建设。西部通道深圳湾公路大桥合龙，福田口岸基础设施建设加速推进。

　　 （四）努力解决瓶颈制约，资源能源保障能力进一步增强。东江水源二期、大鹏半岛支线等一批水源工程进展顺利，北线引水工程已具备全面开工条件。颁布《深圳市节约用水条例》，加大节水宣传，强化节水措施，特区内用水首次实现负增长，全市供水增幅仅为3.3%，大大低于GDP增长速度。加强电网和电力生产建设，东部电厂一期工程、LNG接收站等重大项目顺利推进。积极争取省电力部门的支持，加强与贵州省的电力合作，大力推进节约用电，最大限度地保障了生产生活用电需求。积极应对国际油价上涨带来的影响，协调各方，有效缓解了能源供给紧张状况。

　　 （五）加强改革的统筹协调，综合配套改革稳步推进。国有企业和国有资产管理体制改革在全国处于领先地位。制定实施第三轮市区财政体制调整方案，完善了与事权相适应的财力分配机制。行政、文化、人事等体制改革取得阶段性成果。出台人口管理“1＋5”文件，积极探索与城市经济社会发展相适应的人口管理体制。推进投融资体制改革，与香港地铁公司签署轨道交通4号线特许经营权协议。改革完善工程招投标制度，实施政府投资工程预选承包商制度。行业协会组织机构民间化工作基本完成，政府与行业协会的新型关系初步确立。

　　 （六）对外开放水平不断提高，区域经济合作更加密切。全年实际引进外商直接投资29.69亿美元，增长26.3%。世界500强企业新批和增资项目40个。高新技术产品出口达470.9亿美元，占全市出口总额的46.4%。制定《深圳市应对入世后过渡期行动纲要》，建立进出口与产业损害预警系统。“走出去”步伐进一步加快，海外市场拓展成效明显。对外承包工程业务合同额、营业额分别增长109.5%、64.1%。提出“向香港学习，为香港服务”的理念，进一步加强深港在城市规划、基础设施建设、口岸通关等方面的沟通合作。与周边地区和内地省市的区域经济合作进一步加强。

　　 （七）加强城市规划建设管理，突出解决了一些城市发展难题。召开首次全市规划工作会议，基本完成《深圳2030城市发展策略研究》和宝安、龙岗八大组团分区规划，出台全市整体交通规划和公共交通规划，加强基本生态控制线规划管理。举办首届深圳城市/建筑双年展。坚持“一次性转地、一次性付款、一年内完成”的原则，基本完成了宝安、龙岗两区城市化转地工作，土地利用和供应计划管理得到加强。全面遏制违法抢建、违法用地、违法开发等行为，在刹住违法建筑增量上实现历史性突破。插花地危险边坡、国有预留地、待建地、街道两旁绿化用地上的违法建筑基本清拆完毕。城中村改造工作有序推进。集中开展“清无”行动，超过96%的无证无照非法经营户得到有效治理。

　　 （八）高度重视社会治安、维护稳定和安全生产等工作，城市的安全环境和社会秩序得到有效保障。加强社会治安综合治理，实施科技强警，扩大网格化布警范围，2005年刑事案件同比下降21.2%，特别是危害大、群众反映强烈的严重刑事案件下降31.5%，“两抢”案件下降37.6%。城市应急能力显著增强，依法妥善处置多起重特大突发性事件，较好地处理西部通道侧接线工程、油气供应紧张、停车场收费调整等社会广泛关注的问题和一批群体性上访事件，维护了社会稳定。加强安全生产工作，强化政府部门安全管理责任制，开展全市安全隐患排查整治和安全生产大检查，各类安全生产事故总起数、死亡人数、受伤人数及直接经济损失等四项指标同比都有较大幅度下降。

　　 （九）民主法制建设得到加强，基层基础工作扎实推进。顺利办结路隧收费、机动车尾气治理、生态环境保护等3件市人大议案，抓紧办理新交办的食品安全议案，办复市人大建议555件，政协建议案3件、提案593件。自觉接受人大的法律监督、工作监督，接受人民政协、民主党派的民主监督和新闻媒体的舆论监督。对于涉及城市长远发展和市民重大利益的事项，认真听取各方意见和建议，严格履行听证、公示程序。完善行政许可实施配套办法，实现了全市行政许可实施法定化。通过依法行政和法制教育，企业和市民的责任意识得到加强。基层组织建设和社区建设深入推进，盐田区社区治理体制改革获第三届“中国地方政府创新奖”。加强社区维稳，人民调解的“六约模式”受到国家司法部表彰。

　　 （十）贯彻落实“责任风暴”“治庸计划”，行政执行力建设取得成效。制定实施《关于推进行政管理创新加强政府自身建设的实施意见》、《关于健全行政责任体系加强行政执行力建设的实施意见》及相关配套文件，加强行政问责和督查督办，初步形成了一套比较科学、严密、可操作的行政责任体系。全面加强政府预算管理、财务管理和投资管理。对预算执行和其他财政收支审计工作报告中发现的问题进行严肃查处和整改。强化行政效能监督，创新行政监察方式，运用电子监察系统对行政许可项目进行实时监督。市行政服务大厅集中、公开、高效的办公模式取得显著成效，有力推动了行政管理创新和服务水平的提高。

　　 各位代表，回顾过去五年的发展历程，我们深感成绩来之不易。五年来，我们始终坚持在市委的统一领导下开展工作，紧紧依靠和凝聚全市人民的力量，形成了心往一处想、劲往一处使，团结和谐的工作局面。始终坚持把优化经济结构作为实现经济增长方式转变的中心环节，努力提高支柱产业发展水平，在保持较快发展的同时，不断提高经济效益和质量。始终坚持把改革创新作为发展的不竭动力，大力推进行政管理体制、市场经济体制、社会管理体制的改革创新。始终坚持把维护人民利益作为工作的出发点和落脚点，切实让人民群众分享到经济社会发展的成果。所有这些，是过去5年深圳取得突出成就的集中体现，也是未来深圳再创辉煌的宝贵财富。

　　 各位代表，历届市委、市政府团结带领全市人民，用辛劳和智慧、激情与拼搏，谱写了深圳经济特区发展史上又一精彩篇章。今天的深圳，正在成为一座经济繁荣、环境优美、社会和谐、充满青春活力与创新激情的现代化国际化城市。在此，我代表深圳市人民政府，向奋斗在各条战线的全市广大干部群众和外地来深建设者，向各位人大代表、政协委员、各民主党派、工商联和社会各界人士，向中央和各省区市驻深单位，驻深解放军、武警官兵，向所有关心支持深圳建设的港澳同胞、台湾同胞、海外侨胞和国际友人，表示崇高的敬意和衷心的感谢！

　　 我们也清醒地看到，我市经济社会发展还存在着一些亟待解决的困难和问题，突出表现在：一是转变经济增长方式任务相当艰巨，空间不足、资源能源短缺等问题比较突出。二是人口机械增长过快，人口结构很不合理，城市管理和经济社会发展面临巨大压力。三是涉及群众切身利益的一些问题还没有得到很好解决，城市交通、环境和社会治安等问题仍然比较突出。四是维护社会稳定、构建和谐社会的任务还相当艰巨。五是政府管理和服务水平有待进一步提高，行政执行力还不够强。这些问题，我们将认真对待，采取有效措施切实加以解决。

　　 二、“十一五”时期的指导思想和目标任务

　　 根据《中共深圳市委关于制定深圳市国民经济和社会发展第十一个五年规划的建议》，“十一五”期间我市经济社会发展的指导思想是：以邓小平理论和“三个代表”重要思想为指导，以科学发展观统领经济社会发展全局，以建设和谐深圳效益深圳和现代化国际化城市为总目标，以实施自主创新主导战略为核心，以改革开放为动力，坚持以人为本，坚持抓好发展第一要务，坚持紧约束条件下新的资源观，强力发展循环经济，进一步优化经济结构，创新发展模式，提高发展质量，保护生态环境，推动社会事业全面进步，确保在2010年基本实现社会主义现代化。

　　 到2010年，深圳经济社会发展主要预期目标和约束指标是：

　　 ——本市生产总值年均增长13%左右，2010年达到9000亿元，人均GDP达到1.2万美元。

　　 ——高新技术、金融、物流、文化产业增加值占GDP的比重分别达到35%、15%、12%和10%以上，第三产业比重提高到50%。

　　 ——全社会研究与开发支出占GDP的比重达到3.6%，自主知识产权高新技术产品产值占高新技术产品产值比重提高到65%。

　　 ——万元GDP能耗、万元GDP水耗、万元GDP建设用地分别比2005年下降10%、20%和40%以上，再生资源回收利用率达到98%，城市污水处理回用率达到20%。

　　 ——户籍人口登记失业率控制在3%以内，社区卫生服务人口覆盖率达到95%以上，亿元GDP安全事故死亡率比2005年下降45%。

　　 “十一五”时期，我们要全面落实科学发展观，解决好发展与自然系统的矛盾，在发展循环经济、优化产业结构、建设效益深圳上取得突破性进展；解决好发展与社会系统的矛盾，在实现以人为本、建设和谐深圳上取得突破性进展；解决好规划建设管理中的突出问题，在提升城市综合竞争力、建设国际化城市上取得突破性进展；解决好城市可持续发展的内在动力问题，在实施自主创新主导战略、建设国家创新型城市上取得突破性进展。实现上述目标，必须正确把握好四个关系：

　　 一是经济发展中“快”与“好”的关系。科学发展观的实质是既快又好地发展。完成“十一五”时期的各项目标任务，更好地改善人民生活，要求保持一个较快的发展速度。同时，必须切实转变资源观，在紧约束下求发展，切实转变经济增长方式，大力发展循环经济，把发展循环经济作为全面落实科学发展观、建设效益深圳的重要手段，实现“有效益的速度”和“有速度的效益”的有机结合。

　　 二是社会管理中“宽”与“严”的关系。建设以人为本的和谐社会，需要依法保护市民的合法权益，努力创造一个宽松和谐、安定祥和、各尽所能、充满活力的社会环境；同时，也需要弘扬法治精神，维护法律权威，加强依法行政，严格城市管理。要在以人为本的基础上做到宽严有度、刚柔相济，实现城市生活的文明法治、活而有序。

　　 三是城市建设中“硬”与“软”的关系。未来5年，是我市加速推进城市基础设施建设、提升城市功能的重要时期。我们在加快完善城市交通路网、机场、港口等硬件建设的同时，要更加突出城市的组织、管理、服务和创新等软件建设，在实现城市管理的“科学、严格、精细、长效”上下大功夫。

　　 四是发展动力上“外”与“内”的关系。对外开放是深圳的优势所在、特色所在。要进一步利用好各种外部资源，全方位提升对外开放的广度和深度，不断提高引进消化吸收再创新的能力和水平。在此基础上，大力推进原始创新和集成创新，加快发展壮大一批具有国际竞争力的创新型民族企业。

　　 各位代表，我们将按照以上指导思想、目标任务和工作思路，与全市人民一起团结奋斗，继续充分发挥深圳经济特区改革开放的“窗口”和“试验田”作用，当好建设中国特色社会主义的示范城市，通过五年的不懈努力，把深圳建设成为一个加快发展、率先发展、协调发展的社会主义现代化城市；一个富于创新活力，具有中国特色、中国风格、中国气派的国际化城市；一个自主创新能力突出、四大支柱产业全面发展、具有较强的示范、辐射、带动作用的经济中心城市；一个在改革开放中先行先试、让人民群众充分享受到改革发展成果、和谐深圳效益深圳彰显强大生命力和影响力的可持续发展的先锋城市！

　　 三、2006年的主要工作

　　 今年是实施“十一五”规划的开局起步之年。市委四届四次全会明确了今年经济社会发展的预期目标：在资源能源消耗水平稳步下降的基础上，本市生产总值增长13%左右，出口增长15%左右，全社会固定资产投资增长6%左右，社会消费品零售总额增长14%左右，地方财政一般预算收入同比增长12%左右。综合考虑目标要求和现实可能性，按照“一个重点、三个突破、四个上台阶、强力推进八项工作”的思路，分层次、分阶段推进全年工作。

　　 一个重点

　　 全面实施《关于实施自主创新战略建设国家创新型城市的决定》，以提升科技创新为主线，全面推动思想观念创新、发展模式创新、体制机制创新、对外开放创新、企业管理创新和城市管理创新。要在打造创新型人才高地、创新型企业高地、创新型产业高地、自主知识产权高地等方面取得新成绩。

　　 大力完善创新环境。构建支持自主创新的配套政策体系。创新科技资源配置方式，提高科技资金使用效益。深化科研事业单位改革，提高科技管理和服务水平。支持深交所办好中小企业板块，推动创业板市场体系建设。推进华南技术产权交易中心融入多层次资本市场，启动百家创新企业上市路线图计划，设立创新企业贷款担保风险代偿机制，为创业投资提供更多的退出渠道，完善创新资金链。加强面向社会、资源共享的研发、技术、检测、科技图书馆、标准信息馆等公共平台建设。繁荣哲学社会科学，积极推动理论创新。大力培育创新文化，鼓励创新活动，发挥创新才能，保护创新成果。

　　 大力提升源头创新能力。促进高等教育跨越式发展。加快推进深圳大学二期扩建，努力把深圳大学办成高水平、有特色、综合性的教学研究型大学；加快深圳职业技术学院新校区和深圳信息职业技术学院新校区建设，创新高等职业教育模式。积极改善大学城办学条件，充分利用名校资源，创新机制和思路，提高大学城教学和科研水平，发挥高校在高级人才培养、创新人才聚集以及基础研究和应用基础研究等方面的突出作用。加强同海内外知名高校、研究院所的合作。积极研究规划新的科研型高等院校和科研院所。

　　 大力实施知识产权战略、标准化战略和品牌战略。增强知识产权保护意识，加大知识产权保护力度，实施“全社会、全过程、全方位”的知识产权战略，建设知识产权强市。建立完善知识产权地方政策法规体系，依法严厉打击侵犯知识产权的违法犯罪行为，进一步发挥知识产权制度对创新的激励和保障作用。出台《深圳市标准化战略实施纲要》，引导和支持企业及行业协会积极参与国际标准、国家标准和行业标准制订，推动自主知识产权与技术标准的结合。鼓励企业积极开展自主品牌经营，打造更多具有国际竞争力的名牌。

　　 大力培养和引进各类创新型人才。实施《产业发展与创新人才奖暂行办法》，进一步增强我市对高层次人才的吸引力。放宽创新型人才入户政策，解决其住房、子女就学等实际问题，建立多种形式的“人才驿站”，建立鼓励创新的资助机制。改革人才管理体制，创新专家工作机制，整合人才市场，推进人才资源开发使用的社会化，调动各类人才的积极性。充分发挥企业家服务机构的作用，健全企业家创业发展的支持服务体系。加强科学普及和创新培训，提高市民科技素质和创新能力。

　　 三个突破

　　 第一，改革实现新突破。深化行政管理体制改革。完善行政管理决策机制，进一步推进决策的民主化、科学化。清理规范非行政许可类审批事项，实现审批的法定化和标准化。建立经常性的审批事项审查和行政许可实施情况定期评估机制，完善相对集中的行政审批机制，积极探索“网上审批”。加快推进重大投资项目审批制度改革，优化程序，再造流程，力争年底前将重大投资项目审批时间压缩到100个工作日以内。深化财政体制和地方税收征管体制改革，优化财政支出结构，完善部门预算制度，在市级行政事业单位全面实行国库集中支付制度。整合政务信息化管理体制，提高“电子政务”和“数字深圳”建设水平。进一步理顺部门间和市、区间的职能分工，重点解决政府系统内部职能交叉、责任不清、事权分离和管理真空等突出问题。稳步有序推进街道行政区划调整，理顺市、区、街道之间的事权划分，继续推动政府职能转变。探索建立行政执法与刑事司法相衔接的机制，最大限度地发挥行政执法的作用。

　　 深化经济体制改革。加快国有经济发展步伐，做强做大一批关键和重要领域的国有控股产业集团。健全国有资产监督管理体制，转换国有企业内部经营机制，完善法人治理结构。探索非经营性国有资产监管体制，剥离行政事业单位的经营性资产，建立行政事业性国有资产的分类分级监管体系。推动集体企业产权制度改革，引导规范社区股份合作企业健康发展，提高经营管理水平和效益。整顿和规范市场经济秩序，完善社会信用体系。继续深化投融资体制改革，拓宽基础产业和基础设施的投融资渠道。

　　 深化社会事业和社会管理体制改革。积极稳妥分类推进事业单位改革，将行使行政管理职能的监督管理类事业单位转变为行政机构，将开发经营类事业单位转制为企业，提高社会公益性事业单位的服务水平。创新事业单位管理方式和运行机制，探索“管办分离”、“以事定费”新模式。开放医疗市场，增加医疗供给，改革公立医疗财政供养办法和医护人员薪酬激励机制，强化医疗价格监管。深化文化市场投入体制和综合执法体制改革，探索公益性文化活动社会化运作新模式。改革公用事业监管体制和公用设施管养体制。深化行业协会改革，更好地发挥行业协会、商会以及中介组织在提供服务、反映诉求和规范行为中的作用。

　　 加强改革的综合协调。切实把改革放到更加突出的位置，提高对改革工作重要性和紧迫性的认识。注重改革决策的科学性，增强改革措施的协调性，提高改革的质量和水平，让人民群众享受到改革的成果，使改革真正得到广大人民群众的拥护和支持。认真实施《深圳经济特区改革创新促进条例》，加大对重要改革的宏观指导和对改革计划完成情况的检查监督，依法推进各项改革。

　　 第二，产业结构优化升级实现新突破。加快发展高新技术产业。加快推进高新技术产业向产业链和价值链的高端延伸。在现代装备制造业和光机电一体化领域引进和扶持一批重大项目，提高制造业适度重型化的水平。启动坝光精细化工园区的基础设施建设，进一步提高高新区建设发展水平，加快推进高新技术产业带的建设发展。按照“整体开发、一步到位”的原则，加快大工业区、光明片区基础设施建设力度，全面提高全市各工业园区综合配套和服务水平。积极推进服装、家具、模具、黄金珠宝等九大传统产业集聚基地建设，促进传统工业的优化升级。大力发展高科技农业、都市农业和海洋产业。进一步提高“高交会”办展水平和实效。

　　 加快建设区域金融中心。积极推进罗湖、福田、南山三大金融聚集区的建设，加快宝安、龙岗和盐田金融业发展。大力吸引和鼓励海内外金融机构在深圳设立分支机构和后台服务中心。支持发展各种新型金融机构，组建综合性金融控股集团，鼓励金融创新，增强金融业的综合实力和整体竞争力。配合中央驻深金融监管机构，加强金融监管，提高风险防范能力。

　　 加快发展现代物流业。加强物流工作的领导和协调力度，加快推进六大物流园区建设，抓紧“空港联动”的申报和前期准备工作。鼓励和支持资源性综合物流企业和第三方物流企业做强做大，大力吸引国内外知名供应链管理企业来深设立国际物流总部、区域配送中心、分拨中心等。完善物流公共信息平台和电子口岸建设，进一步提高口岸通关效率。

　　 加快做大做强文化产业。推进文化产业基地建设，加快打造钢琴之城、图书馆之城、设计之都和动漫基地。鼓励文艺精品创作。积极培育一批大型文化企业集团和文化产品品牌，支持广电、报业、发行集团公司加快发展，推动大芬油画村做强做大，壮大一批“专、精、特、新”的中小文化企业。积极吸引国内外知名文化产业集团来深发展。按照保护与开发并重的原则，稳步推进东部滨海地区的规划建设，抓紧东部华侨城、欢乐海岸、大鹏半岛国家地质公园及深圳湾15公里滨海长廊建设。高水平办好第二届“文博会”，参与筹办好“第二届广东国际旅游文化节”，完善城市国际化旅游环境。

　　 加快发展现代服务业。继续壮大商贸、会展、信息服务等优势产业，积极发展会计、律师等专业服务业，全面提升现代服务业发展水平。抓紧出台支持现代服务业发展的相关配套文件，加快服务业向特区外延伸，努力实现特区内外服务业布局一体化。按照《深圳市商业网点规划（2006－2010）》，促进商业网点全面、均衡发展。制定和实施发展总部经济的扶持政策。积极培育和扩大通信、休闲娱乐、医疗保健等服务性消费热点，切实保障消费者合法权益。

　　 第三，人口管理实现新突破。认真执行人口管理“1＋5”文件，加快建立全面、系统、科学的人口管理制度，完善人口管理领导机制和责任制度。加强人口管理立法，探索实施居住证制度，提高劳动密集型产业的准入门槛。强化人口统计监测，加大人口政策执行的检查监督，完善市、区、街道、社区四级人口综合管理架构，增强基层的人口管理力量，实现人口管理由柔性管理向刚性管理转变，由分散管理向集中联合管理转变，由传统手段管理向信息化手段管理转变，有效遏制人口规模急剧膨胀的态势，使人口机械增长走上稳步可控的轨道，使城市人口全面纳入法治化管理的框架。加强计生工作，继续稳定低生育水平，提高人口素质，优化人口结构，建设人力资源强市。

　　 四个上台阶

　　 第一，基层基础和城市管理工作上新台阶。认真开展“基层基础年”和“城市管理年”活动。继续实施固本强基工程，加大基层社区投入力度，理顺社区治理体制，充实、加强基层力量，做到费随事转、权随责转，实现社会管理重心下移。加快社区工作站建设，完善社区管理服务模式，规范社区管理服务内容，强化社区居民自治和服务功能，推进社区工作专业化。认真执行干部下基层驻（挂）社区制度和领导班子成员挂点包干制度，努力建设“六好”和谐平安社区。

　　 加强生态环境建设和保护。积极创建国家首批生态园林城市，认真做好国家环保模范城市复查迎检工作。全面推进治污保洁工程，重点加强深圳河流域和龙岗河、观澜河等跨界河流的污染整治。年内实施大沙河、福田河、新洲河、盐田河、荔枝湖“四河一湖”生态化改造工程，完成深圳河干流、布吉河、深圳水库排洪河、莲塘河、双界河和洪湖“五河一湖”截污治污及生态化改造前期工作。加快宝安、龙岗两区污水处理厂、配套污水管网和垃圾无害化处理等基础设施建设。启动西丽、南山、滨河污水处理厂等新建和改扩建工程。加大空气污染防治力度，实施机动车尾气排放国Ⅲ标准，加快电厂脱硫和“油改气”工作进度，控制扬尘污染。建立环境安全预防和危机应急体系。对基本生态控制线范围和水源保护区实行最严格的管理。重视湿地和海洋环境保护。大力推进森林公园、郊野公园、市政公园、社区公园建设，年底前新建成3个市级森林公园、2个郊野公园、4个市政公园和50个社区公园。

　　 开展市容环境综合整治。加强以城中村和出租屋为重点的环境整治，积极落实路面清理、沿街建筑“穿衣戴帽”、架空管线整治、消防设施完善、环境美化绿化、户外广告招牌清理整顿等综合整治措施，全面治理非法养犬、乱张贴乱涂写、破坏公共设施等损害城市环境的顽症，实现市容环境的严格、覆盖式管理。加快研究出台处理违法建筑的政策措施，同时继续保持高压态势，查处违法建筑和违法用地“力度不减、标准不改、目标不变”。巩固“梳理行动”和“清无”成果，加强后续管理，建立长效机制。

　　 启动数字化城市管理工程。建设城市管理信息系统和广域网、呼叫系统，建立全面、准确、完整的城市管理部件基础数据库，实现城市管理信息资源的整合与共享。以信息化为手段，全面推行城市网格化管理，实现管理精细化，提高城市综合管理的能力和效率。

　　 第二，规划建设工作上新台阶。加大全市各领域、各层次规划的统筹协调力度，建立科学合理的城市规划体系。精心组织好新一轮城市近期建设规划的编制和实施工作，加强城市近期建设规划与国民经济和社会发展“十一五”规划的衔接。启动城市总体规划的修编工作，完善各层次和各专业规划编制。大力推进特区外法定图则的编制工作。完善城市规划实施机制，加强城市规划的监督检查，探索建立城市规划督查巡视制度。加大特区外基础设施建设投入，以完善城市功能和提高建成区质量为核心，推进奥体新城、光明新城、龙华新城等若干高标准的新城区建设，促进特区内外协调发展。按照城市规划的总体布局，在充分保障人民群众利益的基础上，稳步推进城中村（旧村）改造。认真开展城市设计，精心打造中心区中央商务区，努力提高建筑设计水平。

　　 第三，治安、维稳和安全工作上新台阶。全面落实社会治安综合治理责任制和群防群治措施，强化对城郊结合部、出租屋和旅业的综合治理，加强城中村公共娱乐场所安全整治。大力实施科技强警，推进社会治安电子防控工程建设，改革派出所勤务机制，进一步充实一线警力，提高路面见警率，依法严厉打击严重暴力性犯罪、经济犯罪和路面“两抢”等多发性侵财犯罪，努力增强市民的安全感。认真贯彻实施《信访条例》，依靠法律手段妥善处理信访突出问题及重大群体性事件。加强矛盾纠纷排查，高度重视人民调解工作，健全社会矛盾调处机制，引导群众依法表达诉求。整合应急资源，加强“三防”、民防、气象、边坡治理、森林防火等工作，进一步提高城市应急和抗自然灾害的能力。加强安全监管，落实安全生产责任制，加强安全生产执法检查，强力整治安全隐患。加快现代安全模拟教育基地建设步伐，对广大市民开展安全避险自救教育，加强企业员工安全培训。

　　 第四，实施“走出去”战略上新台阶。一是进一步加大“走出去”支持力度。建立服务与援助体系，帮助企业应对知识产权纷争、不正当竞争及其他商业壁垒，推动企业更好地开拓海外市场。鼓励和引导本土企业在海外投资能源资源和原材料行业，加强能源资源储备。二是进一步优化出口结构，完善外贸出口预警和贸易磨擦应对服务机制，积极引导企业用高附加值、高科技含量的产品和服务去占领国际市场。三是稳步提高现代服务业招商引资水平与规模，大力吸引国际服务业外包业务。四是进一步密切深港合作，打造深港创新圈。五是加强国际间城市交流与合作，努力提升城市的国际知名度与影响力。六是积极参与区域合作。重点抓好深圳与泛珠三角区域在基础设施、能源、经贸、旅游、劳务等领域的合作。积极发展海铁联运、江海联运、国际中转等，力争年底开通至长沙、昆明海铁联运“五定班列”。

　　 强力推进八项工作

　　 第一，强力推进人民群众最关心、最直接、最现实的利益问题的解决。优先发展教育事业。继续加大对教育的投入，加快宝安、龙岗两区基础教育的规划布局，合理确定公共教育资源的服务范围，优化教育资源配置，提高资源使用效率，促进义务教育均衡优质发展。大力发展高中阶段教育，积极发展职业教育，规范发展民办教育。进一步完善国民教育体系，构建终身教育体系，努力建设学习型城市。

　　 大力发展医疗卫生事业。按照“一大一小”同步推进原则，市人民医院外科大楼、市疾控中心、第三人民医院和滨海医院等一批重大医疗卫生设施年内开工建设，加快市眼科医院、康宁医院、药品检验所、市中医院住院大楼等项目的建设进度，新建一批社区健康服务中心。增强我市中医药业自主创新能力，提升中医药医疗服务水平。加快劳务工医疗网点建设，提高基层医院和社区健康服务中心的服务能力和覆盖范围。促进民营医疗市场规范发展。加强医德医风建设，规范医疗和用药行为，进一步解决好看病难、看病贵问题。协助办好世界牙科联盟年会。做好艾滋病、高致病性禽流感等防治工作。

　　 切实做好就业和再就业工作。强化就业和再就业目标管理责任制，落实户籍居民按比例就业政策。大力开发公益性就业岗位，加强对困难群体的就业和再就业援助，建立完善“零就业家庭”灭零工作长效机制。高度重视宝安、龙岗两区城市化后居民的就业问题。完善公共就业服务体系，加强职业培训和技能鉴定。鼓励居民自主创业。加强劳动保障监察执法，规范企业用工行为，坚决打击劳务诈骗、恶意欠薪等违法行为，切实维护劳务工合法权益。推动企业履行社会责任，健全劳动保障信访机制，完善政府、工会和企业三方协商机制，促进劳动关系的和谐。

　　 努力提高居民收入和社会保障水平。提高最低工资标准，建立与经济发展相联系的工资增长机制，确保劳动者享受到发展成果。强化水电气等公用事业价格阶梯机制，保证市民基本生活需要不受影响。进一步完善养老保险制度，健全多层次医疗保障体系，全面推广劳务工医疗保险制度。继续扩大社会保障覆盖面，确保各项社保待遇按时足额发放。加强社会保险基金的监督与管理。稳步提高最低生活保障标准，完善社会救助体系，积极支持老龄工作、残疾人事业和社会福利事业，大力发展慈善事业。

　　 加强食品安全工作。全面强化食品、药品、餐饮卫生监管，加快推进食品安全“五大工程”、“四大体系”建设，严厉打击制售假冒伪劣食品行为，强化食品生产、加工和消费等环节的监管，加快农贸市场改造升级步伐，保障人民群众健康安全。

　　 第二，强力推进循环经济发展。大力推动循环经济发展的理念创新、法制创新、政策创新、体制机制创新、产业创新和文化创新，创造有利于循环经济发展的综合环境。认真实施《深圳经济特区循环经济促进条例》，完善促进循环经济发展的政策法规，系统制定全市循环经济发展规划，探索建立符合科学发展观和循环经济要求的地方统计体系，把循环经济发展的相关工作设定为约束性指标。开展循环经济产业园区和重点产业项目试点工作。按照“存量调整”和“增量优选”的原则，重点支持资源节约和综合利用新技术、新工艺、新设备的研究开发，推动原有产业向生态化方向转型；同时大力培育和扶持一批高标准、规范化的资源节约型企业。按照“减量化、再利用、资源化”的基本原则，制订产业节能降耗目标和规范，对消耗高、污染重、技术落后的工艺和产品逐步实施强制性淘汰制度。建立新型高效的再生资源回收利用体系，积极开展中水利用、污水回用，加快太阳能、生物质能、风能的研究应用，创建节水节能型城市。加强舆论宣传，大力倡导以循环、共生和可持续发展为核心的价值观。抓紧研究开展循环经济试点工作方案，积极争取成为国家循环经济试点城市。

　　 第三，强力推进重大基础设施建设。积极推进功能性基础设施体系建设。今年计划安排205个重大建设项目，年度投资275亿元。加大项目前期工作力度，广开投融资渠道，确保重大建设项目成熟一个上一个。积极推进广深港客运专线广深段、杭福深客运专线厦深段、平湖集装箱中心站和新深圳站等项目建设。加快推进盐田港区三期扩建、大铲湾一期、蛇口港区三期等集装箱码头续建工程，力争今年建成3个集装箱泊位。加快大铲湾二期工程、盐田东港区一期工程、盐田西港区4、5、6号泊位的前期工作。加紧铜鼓航道工程建设，完善港口集疏运系统。积极推进机场飞行区扩建工程，加快陆域形成工程建设。抓紧轨道交通1、4号线续建工程和3号线工程建设，2号线年内动工，积极做好5号线前期工作。抓紧轨道交通三期的规划编制和前期工作。大力推进深圳湾口岸、福田口岸建设和皇岗、文锦渡口岸改造工程。做好重大项目的拆迁安置和交通疏导工作，既要切实维护群众利益，又要确保重大项目的顺利进行。

　　 加快构建覆盖全市的现代化交通路网体系。完成南坪快速路一期、盐排高速、深港西部通道深圳侧接线工程、宝安大道等项目建设。加快推进横坪公路、深盐二通道、福龙－龙大高速、107国道宝安段三期改造工程等项目和205国道（深惠路）改造工程建设。加大南坪快速路二期、丹平快速路、南光高速、沿江高速、东部过境通道等项目的前期工作力度，高水平实施深南大道改造工程。抓好特区外公共停车场规划建设，推进特区内外公交一体化。

　　 第四，强力推进民营经济的发展。全面落实扶持民营经济发展的各项政策，消除影响民营经济发展的体制机制障碍，进一步优化民营经济发展环境。加强对民营企业知识产权、自主品牌等的保护。选择一批成长性好、产品竞争力强、法人治理结构比较完善、拥有核心技术或自主知识产权的行业龙头企业，给予大企业“直通车”服务，并在市场准入、投融资、公共技术平台、信息服务等方面采取更有效的措施，支持其做大做强。

　　 第五，强力推进土地资源集约利用。实施最严格的土地管理制度，实行产业用地的全市统一规划，统一审批，科学布局，集约利用。进一步完善产业用地政策，提高工业用地的投资强度和工业厂房的建筑容积率，提高土地的综合利用效率。进一步扩大工业用地的“招拍挂”范围。认真落实宝安、龙岗两区城市化的转地政策，积极解决特区内外转地中的遗留问题，抓紧做好转地后的土地管理工作。研究制定旧工业区、旧城区改造有关政策，搞好土地二次开发。加大全市地下空间资源的规划、开发和管理力度。科学调控房地产土地供应，加快处置闲置用地，促使存量土地有序进入房地产市场。完善房地产信息系统建设，推进房地产市场信息的公开透明，打击恶意炒作，引导房地产业健康发展。增加经济适用房、廉租房和中低价位普通商品房供应。

　　 第六，强力推进资源能源保障工作。完善城市供水安全保障体系，加强饮用水源水质保护，大力推进东部供水工程二期、东江下矶角梯级开发工程、北线引水工程等水源项目的规划建设，积极开展雨洪利用、海水淡化等前期研究论证，提高水资源供应的应急能力。改革完善水源管理体制，加快推进供水企业重组，整合特区内外供水和原水资源。加紧东部电厂、前湾电厂、抽水蓄能电站等重点电厂项目建设，加快供电基础设施建设步伐，完善保供电督查工作机制和电力应急体系，提高有效供电能力。加强重要物资储备，探索建立成品油储备和应急体系。完成宝安、龙岗两区天然气转换利用工作，实现特区内外同步使用天然气。创新燃气管理体制，强化燃气市场管理，保障燃气供应安全。

　　 第七，强力推进精神文明和各项社会事业建设。加强爱国主义教育和公民道德教育，弘扬社会主义荣辱观。深入开展“关爱行动”，在全社会倡导关爱家园、团结互助、扶贫济困的良好风尚。加强法制宣传教育和公民道德教育，做好法律援助和服务工作，提高全民法律意识，培养法治公民、责任公民。畅通政府与市民的沟通渠道，充分听取市民的意见和建议，积极回应市民的呼声，增强市民对城市的认同感。积极发展文化事业，规划建设科学馆新馆、现代艺术馆、深圳歌剧院，加大宝安、龙岗两区社区文体设施建设力度。继续办好“深圳读书月”等一批品牌文化节庆活动。进一步丰富公益性文化活动，完善对未成年人的公共文化服务。加强物质和非物质文化遗产保护。积极发展体育事业，大力开展群众性体育活动，继续做好大运会申办工作，全力备战第十二届省运会。继续实施“同富裕工程”，研究制定生态补偿政策和办法，认真做好对口扶持工作。积极开展国防安全教育和双拥工作，加强国防动员和人民防空。扎实做好统计、外事、侨务、港澳台务、民族宗教、边防、保密、档案等各项工作。

　　 第八，强力推进政府自身建设。在政府工作中全面推行“目标明确、责任清晰、制度保障、绩效考核”的目标管理，是本届政府适应新的发展阶段需要，提升行政执行力，提高抓落实能力的一个重大举措。大功成于精细。实行目标管理的核心，就是使政府的每一项工作有目标要求，有责任主体，有工作进度，有监督考核，有奖惩机制，有责任追究，实现政府行政管理的精细化、流程化、标准化、协同化。把目标管理贯穿于抓落实的每一个环节，贯穿于政府工作的每一个方面，落实到每一个部门、每一个公务员。

　　 健全行政责任体系。全面落实重大建设项目责任制和政府投资项目责任制。在食品安全、环保、城管、交通、公安等与市民密切相关的部门试行部门责任“白皮书”及人民群众评价制度，加强绩效考核和行政监察。建立健全主体明确、层级清晰、具体量化的岗位责任制，制定职位说明书，明确工作责任和工作规程。落实部门主办责任制和行政执法协调机制，严肃处理各种推诿扯皮、不负责的行为。

　　 加强行政监督监察和责任追究。发挥行政监察机关的统筹作用，整合政府监督资源，全过程、全方位加强对政府部门履职情况的监督问责，形成强大的内部监督压力，确保政令畅通，执行到位。加强行政效能监察和绩效评估，创新监察手段，推进电子政务和电子监察建设，扩大电子监察在政府工作监督中的覆盖面。加强政府督查工作，完善政务督查信息网络，提高督查实效。积极探索层级监督的新思路，创新行政复议制度。大力实施“阳光工程”，积极推进政务公开，完善政府信息网上公开制度。坚持从严治政，切实加强责任追究，加大惩戒力度，形成“闭环效应”。

　　 积极推进依法行政。加快推进政府自身建设的立法工作，努力实现行政行为在每一个环节上的规范化和法定化，以立法形式推动政府管理方式的改革与创新。认真执行《公务员法》、《行政许可法》和《全面推进依法行政实施纲要》，切实落实行政执法责任制和行政执法过错责任追究制度，严格按照法定权限和程序行使权力，履行职责，接受监督，坚决纠正和查处有法不依、执法不严的行为，提高政府依法管理社会的能力。健全政府重大决策及相关文件的法律审查制度，完善政府法律顾问制度，积极利用法律手段解决城市发展过程中的难题，依法推进各项工作。

　　 大力加强政风建设。保持和发扬奋发有为的精神状态和求真务实的工作作风，坚持重实际、说实话、办实事、求实效。艰苦奋斗、厉行节约，加强财政资金管理，扩大财政资金绩效审计，提高财政资金使用效益，严肃查处奢侈浪费的行为。加强公务员队伍建设，完善公务员培训体系，推行公务员行为规范，探索公务员专业化改革，试行聘任公务员制度，建立科学的考核评价指标体系和个性化评价制度。进一步加强廉政建设和反腐败斗争，加强党纪政纪法纪约束，认真开展商业贿赂治理专项工作，坚决纠正损害群众利益的不正之风，严肃查处违反行政纪律和侵害人民群众利益的案件，努力做到“为民、务实、清廉”，在建设有限政府、责任政府、高效政府、法治政府、廉洁政府上迈出更大的步伐。

　　 各位代表，当前，我们正处在黄金发展期和矛盾凸显期相互交织的特殊时期。市政府将大力加强民主法制建设，自觉坚持向市人大及其常委会报告工作制度，接受人大的法律监督和工作监督，充分尊重市政协、各民主党派和社会各界在政治协商、民主监督和参政议政中的作用，认真办理市人大议案、建议和市政协提案、建议案，充分发挥工、青、妇等人民团体的桥梁和纽带作用，高度重视新闻舆论和人民群众的监督，充分听取市民的意见和建议，在全市上下继续营造一个团结干事的良好氛围，调动一切积极因素，形成推进工作的强大合力。

　　 各位代表，深圳未来5年发展的蓝图已经绘就。重任在肩，必须只争朝夕、奋发进取，狠抓落实、毫不懈怠，努力保持昂扬向上、顽强拚搏的精神状态，把全部心思倾注到干事业上，把全部精力集中到办实事上，把功夫真正下到抓落实上，在真抓实干中开拓前进！让我们高举邓小平理论和“三个代表”重要思想伟大旗帜，牢固树立科学发展观，全面贯彻党的十六大和十六届五中全会精神，在党中央、国务院、省委、省政府和市委的坚强领导下，团结带领全市人民，振奋精神，勤勉务实，认真落实市第四次党代会和市委四届四次全会精神，为建设和谐深圳效益深圳和国际化城市，为开创我市“十一五”时期经济社会发展新局面而努力奋斗！
